# Fluorescence and Hyperspectral Sensors for Nondestructive Analysis and Prediction of Biophysical Compounds in the Green and Purple Leaves of *Tradescantia* Plants

**DOI:** 10.3390/s24196490

**Published:** 2024-10-09

**Authors:** Renan Falcioni, Roney Berti de Oliveira, Marcelo Luiz Chicati, Werner Camargos Antunes, José Alexandre M. Demattê, Marcos Rafael Nanni

**Affiliations:** 1 Department of Agronomy, State University of Maringá, Av. Colombo, 5790, Maringá 87020-900, Paraná, Brazil; rboliveira@uem.br (R.B.d.O.); mlchicati@uem.br (M.L.C.); wcantunes@uem.br (W.C.A.); mrnanni@uem.br (M.R.N.); 2 Department of Soil Science, Luiz de Queiroz College of Agriculture, University of São Paulo, Av. Pádua Dias, 11, Piracicaba 13418-260, São Paulo, Brazil; jamdemat@usp.br

**Keywords:** chemometrics, chlorophyll a fluorescence, hyperspectral vegetation indices, partial least squares regression, principal component analysis, optical spectroscopy, UV-VIS-NIR-SWIR

## Abstract

The application of non-imaging hyperspectral sensors has significantly enhanced the study of leaf optical properties across different plant species. In this study, chlorophyll fluorescence (ChlF) and hyperspectral non-imaging sensors using ultraviolet-visible-near-infrared shortwave infrared (UV-VIS-NIR-SWIR) bands were used to evaluate leaf biophysical parameters. For analyses, principal component analysis (PCA) and partial least squares regression (PLSR) were used to predict eight structural and ultrastructural (biophysical) traits in green and purple *Tradescantia* leaves. The main results demonstrate that specific hyperspectral vegetation indices (HVIs) markedly improve the precision of partial least squares regression (PLSR) models, enabling reliable and nondestructive evaluations of plant biophysical attributes. PCA revealed unique spectral signatures, with the first principal component accounting for more than 90% of the variation in sensor data. High predictive accuracy was achieved for variables such as the thickness of the adaxial and abaxial hypodermis layers (R^2^ = 0.94) and total leaf thickness, although challenges remain in predicting parameters such as the thickness of the parenchyma and granum layers within the thylakoid membrane. The effectiveness of integrating ChlF and hyperspectral technologies, along with spectroradiometers and fluorescence sensors, in advancing plant physiological research and improving optical spectroscopy for environmental monitoring and assessment. These methods offer a good strategy for promoting sustainability in future agricultural practices across a broad range of plant species, supporting cell biology and material analyses.

## 1. Introduction

The use of non-imaging hyperspectral or proximal sensors along with chlorophyll fluorescence techniques has made substantial progress in evaluating the biophysical characteristics of leaves and plants. These state-of-the-art methods offer insights into the physiological conditions of plants, facilitating an extensive understanding of their responses to diverse environmental factors [1,2]. In addition to traditional assessment methods, these sensor technologies enable new ways to monitor plant health and detect changes in the biophysical attributes of leaves, aligning structure and ultrastructure components while optimizing sustainable agricultural practices and plant management [3]. The integration of biophysical analyses is important for improving predictive models, enhancing our understanding of plant-environment interactions, and guiding precision agriculture, ultimately contributing to more resilient and sustainable ecosystems.

Chlorophyll fluorescence (ChlF) curves are invaluable resources for gauging plant health by accurately mapping pigment levels and other biochemical properties, such as protein and carbohydrate content [4,5]. These curves measure the fluorescence emitted by chlorophyll a and provide critical information on photosynthetic efficiency and plant stress responses [6,7]. In this sense, the ChlF curve data reveal variations in biophysical properties at the cellular level, which is important for understanding and modeling plant behavior. The ability of this technique to detect early stress indicators before visible symptoms occur makes it particularly effective for proactive agricultural and environmental management and contributes valuable insights to the fields of plant physiology and ecology [8].

Ultraviolet-visible-near-infrared shortwave infrared (UV-VIS-NIR-SWIR) hyperspectral and chlorophyll fluorescence imaging and non-imaging sensors generate comprehensive spectral profiles, capturing detailed plant information across a broad spectrum of wavelengths [9,10,11]. In particular, non-imaging sensors allow swift data collection without a spatial biophysical context, which is highly efficient for large-scale or remote-sensing evaluations [12,13,14]. The precision and accuracy of these sensors for detecting minor physiological changes significantly increase their ability to monitor biophysical responses, as reviewed in detail [6,7].

The cellular and foliar architecture of plants is correlated, with various chloroplastic cells and leaf optical properties, such as thylakoid granum, adaxial hypodermis, chloroplast number, total leaf thickness, and parenchyma thickness [15,16]. Structural components such as the epidermis, hypodermis, and leaf parenchyma are crucial for maintaining the biomechanical integrity of plant tissues. Modifications of these cells and tissue arrangements can differ greatly and are influenced by factors such as leaf area expansion and cellular structural changes due to biophysical compounds [17,18,19,20]. These variations offer significant insights into the adaptive mechanisms of plants under environmental conditions [21,22,23]. Recent research has shown that fluorescence techniques and hyperspectral sensors use different methods to analyze and predict biochemical and biophysical compounds to monitor the biophysical properties of the leaves, steams, and roots of lettuces, tomatoes, and ornamental plants [11,24,25]. These tools assess changes in cellular size and volume correlated with water content and respond to pigment content, indirectly reflecting variations in concentrated pigments such as chlorophylls and carotenoids correlated with chloroplast numbers or thylakoid stacking in cells [26,27,28].

In leaves with biochemical and biophysical variations, optical properties such as reflectance, transmittance, and absorbance are primarily shaped by structural features, including thickness, cell organization, and mesophyll composition [11,29,30,31]. These properties are further influenced by biochemical elements, particularly pigment concentration and type, which affect light absorption and distribution. In *Tradescantia* plants, the use of these three spectral modes along with fluorescence data provides a precise evaluation of the biophysical mechanisms governing light interactions. This approach enhances the prediction of structural traits, such as leaf thickness, while efficiently estimating key biochemical components such as pigments. Additionally, the use of these sensors, along with others, has proven effective in analyzing foliar structural and ultrastructural components with interaction light with matter, or in this example for tissue plants [10,32,33,34,35].

Hyperspectral Vegetation Indices (HVIs) utilize reflectance values across different wavelengths to assess the plant growth status, health, and photosynthetic efficiency. For example, recent advancements have highlighted the value of HVIs in analyzing extensive datasets from both chlorophyll fluorescence and hyperspectral measurements [36,37,38,39]. For example, HVIs simplify complex spectral data into manageable indices, facilitating large-scale monitoring of biophysical and physiological conditions. These indices can identify regions and bands in which key biophysical components show significant changes. HVIs can detect variations in chlorophyll content, water stress, and nutrient levels [7,40,41]. When combined with chlorophyll fluorescence data, HVIs enhance the ability to differentiate areas with varying photosynthetic rates or stress responses, enabling precise mapping of biophysical markers across landscapes [9,42,43].

Principal Component Analysis (PCA) is a powerful statistical method used to handle the complex, high-dimensional nature of hyperspectral and fluorescence data [11,43]. This technique effectively reduces data dimensionality while retaining most of the variance and uncovering the underlying patterns within the samples. PCA is particularly useful for determining the optimal number of principal components needed to capture essential data features and balancing information retention with noise reduction in datasets. The β-loadings from PCA clearly highlight spectral peaks, revealing the “fingerprints” of physical properties in the dataset, which are correlated with many biophysical parameters [11,24,44,45]. By combining PCA with regression coefficients from various spectral curves, such as OJIP and hyperspectral reflectance, transmittance, and absorbance curves, a more comprehensive analysis integrating biochemical and biophysical insights is possible using prediction models [17,28,46].

The second main method of development is Partial Least Squares Regression (PLSR), a robust method for predictive modeling with hyperspectral data that can manage highly collinear and multivariate datasets. PLSR projects predictor variables (spectral bands) onto a new subspace defined by latent variables that account for the predictor variance and their correlation with the response variable. This method is particularly beneficial when the number of predictors exceeds the number of observations [11,36,47]. PLSR effectively addresses multicollinearity among spectral bands, reducing predictors to a smaller set of uncorrelated components, while capturing key response variable patterns. Combining PLSR with cross-validation techniques enhances model stability and predictive performance, making it the preferred choice for handling complex, large hyperspectral datasets [11,36,47].

Machine Learning (ML), Deep Learning (DL), and Artificial Intelligence (AI) models have made significant contributions to biophysical analysis, particularly in predicting and selecting the most relevant wavelengths for hyperspectral data [2,36,48]. For example, random forests and Convolutional Neural Networks (CNNs) have been successfully applied to detect subtle variations in plant health and stress indicators, whereas Support Vector Machines (SVMs) [49] have been effective in classifying spectral data [50]. These advanced techniques allow automated feature selection and can identify critical spectral regions without human intervention. Despite these advancements, PLSR models have proven to be efficient, offering high predictive accuracy and robust performance in scenarios where interpretability and computational simplicity are key, making them a reliable choice alongside ML and DL models [37,43,51,52,53].

**Figure 1:**
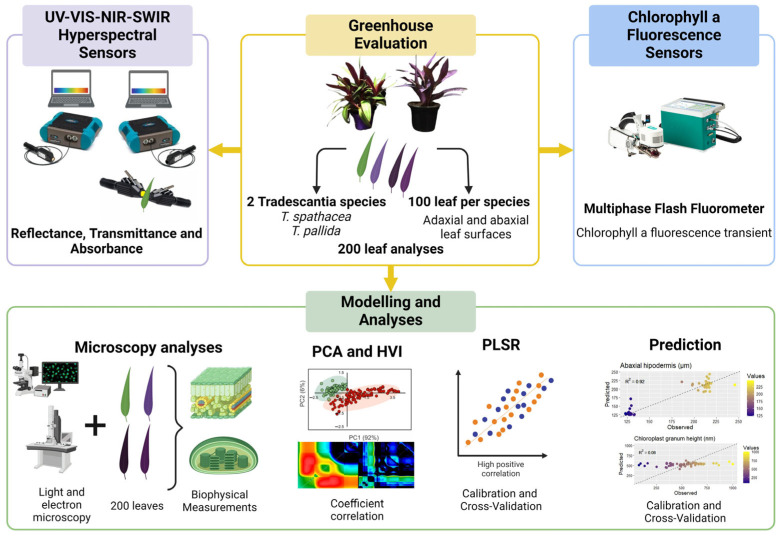
Flowchart for predicting biophysical compounds.

This study focused on improving the prediction of biophysical parameters using chlorophyll fluorescence kinetics and hyperspectral sensors in two *Tradescantia* species. The primary objective was to identify spectral bands in hyperspectral data that correlate strongly with chlorophyll a fluorescence, aiding in the estimation of eight biophysical parameters in green and purple *Tradescantia* leaves. By integrating advanced hyperspectral sensors for reflectance, transmittance, and absorbance data with fluorescence kinetics and utilizing multivariate statistical methods, such as PCA and PLSR, we hypothesized that the prediction accuracy for these parameters could be significantly enhanced, even with variations in leaf coloration (Figure 1).

## 2. Material and Methods

### 2.1. Plant Materials and Experimental Analysis

The species of *Tradescantia spathacea* and *Tradescantia pallida*, which were chosen for their unique biophysical characteristics, were grown in 2-L pots at the Botanical Garden affiliated with the State University of Maringá, Brazil. The conditions within the greenhouse included exposure to natural light, with temperatures ranging from 22 to 26 °C and a prolonged light period of 16 h. The irrigation schedule was set to twice a day, once at 8 a.m. and then again at 6 p.m., to ensure regular soil hydration. For a thorough evaluation, leaf samples were harvested from different sections of the plant. This research aimed to conduct an in-depth hyperspectral reflectance analysis and detailed biophysical examination of 200 leaf samples, the results of which are presented in Figure 1.

### 2.2. Chlorophyll a Fluorescence Measurement

Chlorophyll a fluorescence was measured using an infrared gas exchange analyzer (IRGA) paired with a Multiphase Flash™ Fluorometer (LI-6800-01; LI-COR Inc., Lincoln, NE, USA). The sensor detects chlorophyll fluorescence at wavelengths greater than 720 nm. Leaves were dark-acclimated for 12 h before being subjected to a saturating light pulse of 15,000 µmol m^−2^ s^−1^ for 1 s to induce chlorophyll a fluorescence, ensuring the closure of all reaction centers [11,29].

### 2.3. Hyperspectral Optical Properties of Leaves

The reflectance and transmittance properties of the leaves were simultaneously measured using a FieldSpec^®^ 3 spectroradiometer with an ASD Contact PlantProbe^®^ (ASD Inc., Boulder, CO, USA). The spectroradiometer featured a VNIR detector with a 512-element silicon array for the 350–1000 nm range, a SWIR 1 detector with a graded index InGaAs photodiode for the 1001–1800 nm range, and a SWIR 2 detector for the 1801–2500 nm range. The standard white reference plates were used for calibration. During the measurements, a high-intensity light beam was directed at the adaxial leaf surface, and reflectance and transmittance were recorded. The absorbance was calculated using the formula A = 1 − (R + T), ensuring precise measurements across a broad wavelength spectrum [11,29].

### 2.4. Sample Preparation and Microscopy

#### 2.4.1. Sample Preparation for Microscopy

The leaf samples were prepared for optical microscopy (OM), transmission electron microscopy (TEM), and scanning electron microscopy (SEM). The samples were cut into small pieces, quickly submerged in a fixative solution of 2.5% glutaraldehyde and 2% paraformaldehyde in 0.05 M cacodylate buffer (pH 7.2), and left for six hours. The samples were fixed with a solution of 1% osmium tetroxide and 1.6% potassium ferrocyanide in the same buffer. After fixation, the samples were contrasted with 0.5% uranyl acetate for 12 h and dehydrated with a graded acetone series. Some samples were reserved for SEM, whereas others were embedded in Spurr low-viscosity epoxy resin and sectioned using an ultramicrotome [11,29].

#### 2.4.2. Optical Microscopy

Leaf sections, 1 μm thick, were stained with 1% toluidine blue in borax buffer and briefly heated to enhance staining. A Leica ICC50 optical microscope (Leica Inc., Buffalo Grove, IL, USA) was used to observe the anatomical features, and quantitative analysis was performed using ImageJ (https://imagej.nih.gov/ij, accessed on 20 April 2024) and Image-Pro Plus version 4.5 software (Media Cybernetics Inc., Rockville, MD, USA) [11,29].

#### 2.4.3. Scanning Electron Microscopy (SEM)

The samples prepared for SEM were dried using the CPD-030 Bal-Tec dryer (Bal-Tec AG, Balzers, Liechtenstein, Germany) critical-point drying method, mounted on aluminum stubs with carbon tape, and coated with gold. Observations were made using a Quanta 250 scanning electron microscope (FEI Company, Hillsboro, OR, USA) operating at 15–20 kV, and quantitative analysis was conducted using Image-Pro Plus version 4.5 software (Media Cybernetics Inc., Rockville, MD, USA) [11,29].

#### 2.4.4. Transmission Electron Microscopy (TEM)

Ultrathin sections (60–70 nm) using an ultramicrotome (MTX Powertome X, Boeckeler Instruments, RMC Products, Phoenix, AZ USA) were placed on copper grids, contrasted with 3% uranyl acetate and lead citrate, and observed under a JEOL JEM-1400 transmission electron microscope Leica Microsystems Inc., Buffalo Grove, IL, USA) at 80 kV. A detailed examination of the cellular ultrastructures was performed, and the data were analyzed using the Image-Pro Plus version 4.5 software (Media Cybernetics Inc., Rockville, MD, USA) [11,29].

### 2.5. JIP Test and Hyperspectral Parameters Based on Hyperspectral Vegetation Indices with Optimal Wavelengths

To assess whether selecting the two most responsive hyperspectral wavelengths could enhance the predictive accuracy of eight biophysical parameters, including adaxial epidermis thickness (μm), adaxial hypodermis thickness (μm), parenchyma thickness (μm), abaxial hypodermis thickness (μm), total leaf thickness (μm), chloroplast count, granum height (nm), and thylakoid layer thickness, we examined all potential combinations of two spectral bands using the normalized difference vegetation index formula, as proposed by Crusiol et al. (2023) [51]. Each combination, representing a unique hyperspectral vegetation index (HVI), was correlated with leaf structural and ultrastructural parameters through Pearson’s correlation coefficient (r) and the coefficient of determination (R^2^), using a custom-written IDL code. Hyperspectral data were collected using a ground-based sensor covering the full spectral range of 350–2500 nm [25,29]. The resulting matrices were visualized as contour maps. Additionally, the reflectance, transmittance, and absorbance data for the selected wavelengths were analyzed, as were the fluorescence responses from 0 to 1 s, expressed through OJIP curves captured by a fluorescence sensor.

(1) $$HVI=\frac{Wavelength\;1-Wavelength\;2}{Wavelength\;1+Wavelength\;2}$$

### 2.6. Statistical Analyses

#### 2.6.1. Descriptive, Univariate, and Multivariate Statistical Analyses

Descriptive statistics included count, mean, median, minimum, maximum, and coefficient of variation (CV). Pearson’s correlation was used to explore the relationships between the biochemical and biophysical attributes. Statistical analyses were performed using Statistica 10^®^ (StatSoft Inc., Tulsa, OK, USA) and R statistical package (R Core Team, 2020), with graphical representations created using SigmaPlot 10.0 (Systat Software, Inc., San Jose, CA, USA), Excel 365 software (Microsoft Office Professional 2024, Sunnyvale, CA, USA), and CorelDraw 2020^®^ editor (Corel Corp., Ottawa, ON, Canada).

#### 2.6.2. Principal Component Analysis (PCA)

PCA reduces the dimensionality of hyperspectral data by identifying the principal components that capture the most variance within a dataset. This method effectively filters out noise and highlights the most relevant spectral features, enabling more accurate analysis of biophysical parameters without overfitting or underfitting the model [53,54,55,56,57,58].

#### 2.6.3. Spectroscopy Data Analysis via Partial Least Squares Regression (PLSR)

The hyperspectral data were mean-centered and analyzed using PLSR to develop prediction models for JIP test parameters. The data were split into training (75%) and prediction (25%) datasets. Calibration and cross-validation were used to assess model performance, with metrics such as R^2^, RMSE, and RPD used to evaluate the quality, precision, and accuracy [59].

## 3. Results

### 3.1. Statistical Description of the Biophysical Compounds

Table 1 shows the results of the descriptive statistical analysis of various biophysical compounds measured in the leaves of *Tradescantia spathacea* and *Tradescantia pallida*, with 200 samples per parameter (Table 1).

The descriptive analyses revealed that the mean thickness of the adaxial epidermis was 102.7 μm, the median thickness was 88.0 μm, the minimum thickness was 61 μm, and the maximum thickness was 162 μm, with a coefficient of variation of 23.94%. The adaxial hypodermis thickness had a mean of 323.4 μm, a median of 325.7 μm, a minimum of 212 μm, and a maximum of 452 μm, with a coefficient of variation of 25.79%. The parenchyma thickness had a mean of 252.6 μm, a median of 251.3 μm, a minimum of 224 μm, and a maximum of 296 μm, with a coefficient of variation of 3.80%. The abaxial hypodermis thickness had a mean value of 170.5 μm, a median value of 164.7 μm, a minimum value of 117 μm, and a maximum value of 246 μm, with a coefficient of variation of 25.53%. The total leaf thickness had a mean of 849.1 μm, a median of 820.5 μm, a minimum of 735 μm, and a maximum of 978 μm, with a coefficient of variation of 7.02% (Table 1).

The mean number of chloroplasts per cell was 21.2, with a median of 20.5, a minimum of 14, and a maximum of 32, with a coefficient of variation of 22.23%. The mean granum height was 522.5 nm, the median height was 531.9 nm, the minimum height was 75 nm, and the maximum height was 1000 nm, with a coefficient of variation of 37.29%. The thickness of the thylakoid layers within the granum had a mean of 25.2 nm, a median of 26.0 nm, a minimum of 4.0 nm, and a maximum of 53.0 nm, with a coefficient of variation of 33.80% (Table 1).

### 3.2. Cluster Heatmap of Biophysical Parameters in Tradescantia Species

**Figure 2:**
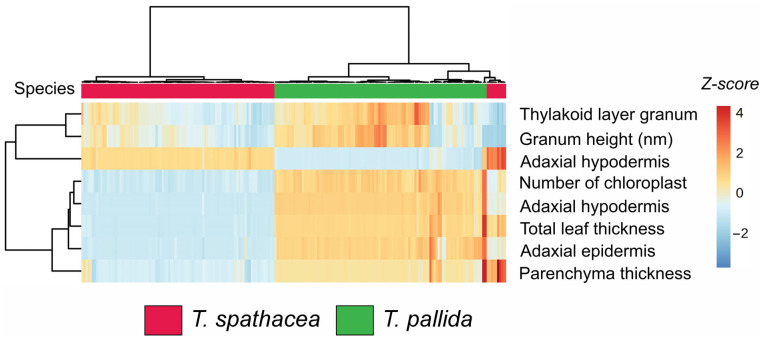
Cluster heatmap of biophysical parameters in leaves. Red represents *Tradescantia spathacea,* and green represents *Tradescantia pallida*. (*n* = 100).

The hierarchical clustering heatmap (Figure 2) shows the biophysical parameters of the leaves of two species: *Tradescantia spathacea* (indicated in red) and *Tradescantia pallida* (indicated in green). The heatmap visualizes the Z-scores for various measured parameters, facilitating the comparison and identification of patterns across species (Figure 2).

The clustering dendrogram revealed distinct groupings. Specifically, *T. spathacea* presented greater Z-scores (indicated by more intense red hues) for parameters such as thylakoid layer granum, granum height, and adaxial hypodermis, suggesting that *T. spathacea* has more substantial structural attributes than *T. pallida* (Figure 2). Conversely, *T. pallida* displayed lower Z-scores (shades of blue) for these parameters, indicating relatively reduced biophysical characteristics.

Furthermore, the number of chloroplasts and total leaf thickness varied significantly among the species. *T. spathacea* tended to have a greater number of chloroplasts and greater leaf thickness, which could imply a greater photosynthetic capacity and structural robustness. The parenchyma thickness and adaxial epidermis also differed notably, with *T. pallida* exhibiting thicker parenchyma and adaxial epidermis, potentially contributing to its adaptive traits (Figure 2).

### 3.3. Analysis of Chlorophyll a Fluorescence and Spectral Properties

Compared with that of *T. pallida*, the chlorophyll a fluorescence of *T. spathacea* was distinctly changed (Figure S1A). The fluorescence measurements for *T. spathacea*, shown with green lines, revealed higher yields than the red–pink lines, which represent *T. pallida* at multiple observation points. We examined several parameters, including the initial fluorescence (O), intermediate phases (J, I), and peak (P). *T. spathacea* consistently presented elevated values, indicating differences in photosynthetic competency and light absorption capacity (Figure S1). The difference in fluorescence yield was statistically significant, as evidenced by the values of F = 230.85, accuracy (Acc) = 0.98, and Kappa (K) = 0.96 (refer to Figure S1A).

The reflectance spectra showed considerable variations between the two species at specific wavelengths. In the UV–VIS spectrum (350–700 nm), a notable difference was observed at 544 nm, where *T. spathacea* had a higher reflectance. The most significant difference is observed at 700 nm in the NIR range (700–1300 nm). In the SWIR1 spectrum (1300–1800 nm), the key difference appeared at 1441 nm, and in the SWIR2 range (1800–2500 nm), it appeared at 2488 nm. These results were statistically significant, with F = 58.71, Acc = 0.90, and K = 0.80, underscoring the importance of these wavelengths for species differentiation based on reflectance properties (Figure S1B).

Transmittance measurements revealed significant differences at specific wavelengths. In the UV–VIS spectrum, the largest difference was observed at 400 nm. In the NIR spectrum, it was 1125 nm, in the SWIR1 spectrum at 1607 nm, and in the SWIR2 spectrum at 2175 nm. These results, with low p-values, suggest the potential of using transmittance at these wavelengths to distinguish between the two species. The transmittance data were characterized by F = 47.17, Acc = 0.85, and K = 0.70 (Figure S1C).

The absorbance measurements revealed notable differences at certain wavelengths across the various spectral ranges. Some wavelengths were 495, 498, 733, and 800 nm in the NIR region, 1575 nm in the SWIR1 region, and 2175 nm in the SWIR2 region. The low p-values confirmed the statistical importance of these wavelengths, indicating their utility in distinguishing between *T. spathacea* and *T. pallida*. The absorbance data revealed F = 87.94, Acc = 0.97, and K = 0.94 (Figure S1D). Overall, the differences observed in the reflectance, transmittance, and absorbance spectra highlight the distinct optical properties of the two *Tradescantia* species (Figure S1).

### 3.4. Principal Component Analysis of the Spectral Data

**Figure 3:**
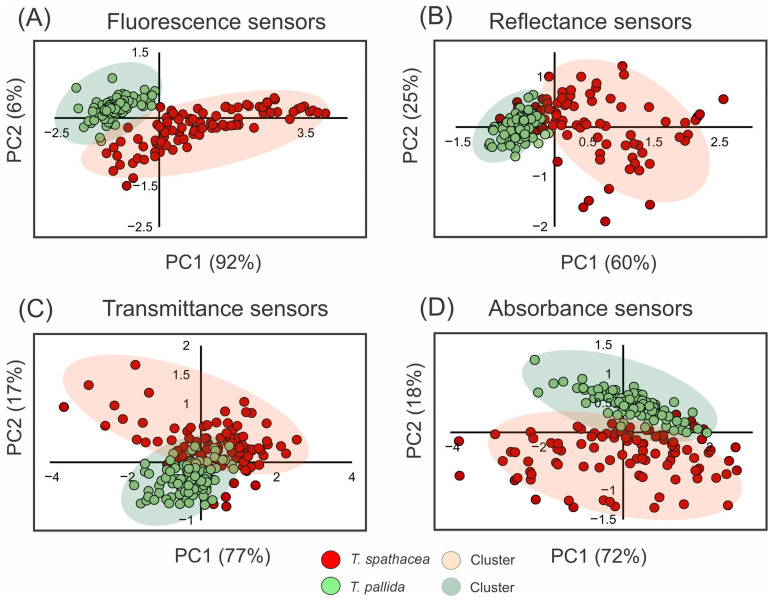
Principal component analysis (PCA) of spectral data from 350 to 2500 nm. (**A**) Fluorescence sensor data. (**B**) Hyperspectral reflectance sensor data. (**C**) Hyperspectral transmittance sensor data. (**D**) Hyperspectral absorbance sensor data. Clusters are indicated by red circles for *T. spathacea* and green circles for *T. pallida*. (*n* = 100).

Figure 3 shows the use of principal component analysis (PCA) to determine the spectral properties of the leaves of *T. spathacea* and *T. pallida* via fluorescence, reflectance, transmittance, and absorbance readings (Figure 3A–D). For example, Figure 3A shows the PCA results for the fluorescence sensors, with *T. spathacea* indicated by red dots and *T. pallida* indicated by green dots. The first principal component (PC1) accounted for 92% of the variability, clearly distinguishing the two plants based on their fluorescence signatures, whereas the second principal component (PC2) explained 6% of the variability (Figure 3).

The PCA of the reflectance data in Figure 3B shows a clear separation along PC1, which accounts for 60% of the variation, and PC2 elucidates an additional 25%, demonstrating unique reflectance characteristics between the species. The PCA results are depicted in Figure 3C, revealing a marked contrast between *T. spathacea* and *T. pallida* along both PC1 and PC2, with PC1 accounting for 77% of the total variance and PC2 contributing 17%, indicating differential species-specific transmittance traits. Figure 3D shows the PCA related to the absorbance sensors, with PC1 representing 72% of the variation and PC2 representing another 18%. These data suggest significant differences in absorbance across species.

The shaded areas in each plot represent the clustering of individual observations showing intraspecific variability. Overall, the PCA results demonstrated that these spectral assessments were effective for differentiating *T. spathacea* from *T. pallida,* as shown in Figure 3.

**Figure 4:**
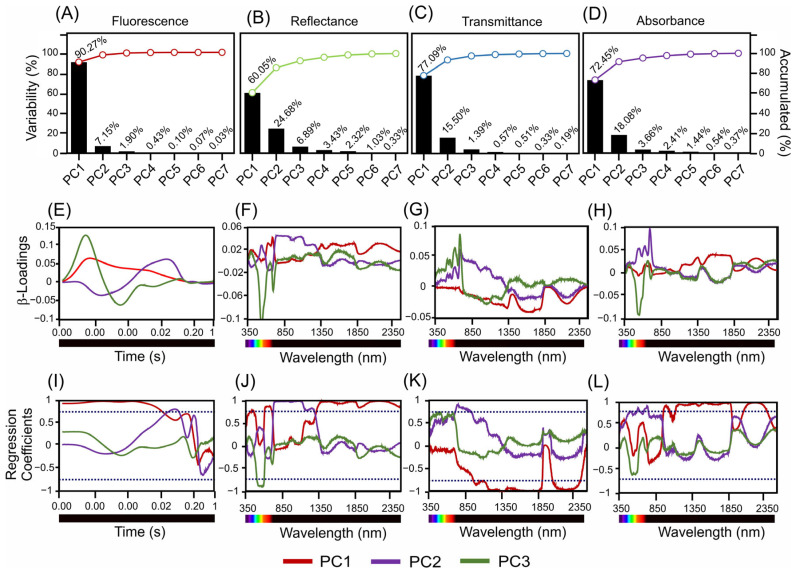
Present scores, β-loadings, and regression coefficients derived from sensors. (**A**–**D**) Variability and cumulative percentages are explained by three principal components. (**E**–**H**) β-Loading associated with hyperspectral and fluorescence sensors. (**I**–**L**) Regression coefficients for PC1–3 in the 350–2500 nm range. The dark blue dotted lines indicate −0.70 and 0.70 correlation of coefficients.

Figure 4 shows an in-depth analysis of the variance, β-loading, and regression coefficients across the different spectral measurements for fluorescence, reflectance, transmittance, and absorbance in the two *Tradescantia* species. This figure shows the contribution of the principal components to the interpretation of spectral data.

Figure 4A–D depicts the variance explained by the principal components for each type of spectral measurement. The fluorescence data (Figure 4A) revealed that PC1 accounted for 90.27% of the variance, with the subsequent components contributing significantly less. For the reflectance data (Figure 4B), PC1 explained 60.05% of the variance, while PC2 explained 24.68%. According to the transmittance data (Figure 4C), PC1 captured 77.09% of the variance, while PC2 accounted for 15.50%. The absorbance data (Figure 4D) indicate that PC1 explained 72.45% of the variance, with smaller contributions from PC2 (18.08%).

The β-loading plots (Figure 4E–H) show the impact of each principal component across the time or wavelength range, highlighting the influence of specific spectral bands. For the fluorescence sensors (Figure 4E), the β-loadings of PC1, PC2, and PC3 showed significant contributions across the measured time points. Reflectance data (Figure 4F) identified key wavelengths for PC1 in the blue, green, red, NIR, SWIR1, and SWIR2 bands, with similar influential wavelengths for PC2 and PC3. The transmittance data (Figure 4G) highlight the crucial wavelengths for PC1 in the blue and SWIR2 bands, with consistent influential spectral bands for PC2 and PC3. The absorbance measurements (Figure 4H) for PC1 indicated key wavelengths spanning from the blue to SWIR2 range, which is consistent with the results for PC2 and PC3.

The regression coefficient plots (Figure 4I–L) further illustrate the influence of each wavelength on the respective spectral measurements, identifying the most impactful wavelengths. The regression coefficients for fluorescence (Figure 4I) confirmed a significant influence across the measured time-points. The reflectance data (Figure 4J) underscore the importance of the blue, green, red, NIR, SWIR1, and SWIR2 bands, whereas the transmittance data (Figure 4K) highlight the wavelengths in the blue and SWIR2 bands. The absorbance data (Figure 4L) validated the relevance of wavelengths ranging from blue to SWIR2.

Overall, the detailed analysis in Figure 4 highlights the significant contributions of specific wavelengths and spectral bands to the variance in the fluorescence, reflectance, transmittance, and absorbance measurements. This result showed the most influential spectral regions for distinguishing *T. spathacea* from *T. pallida*, providing valuable insights for further ecological and physiological research (Figure 4).

### 3.5. Evaluation of Hyperspectral Vegetation Indices via Fluorescence, Reflectance, Transmittance, and Absorbance Sensors

**Figure 5:**
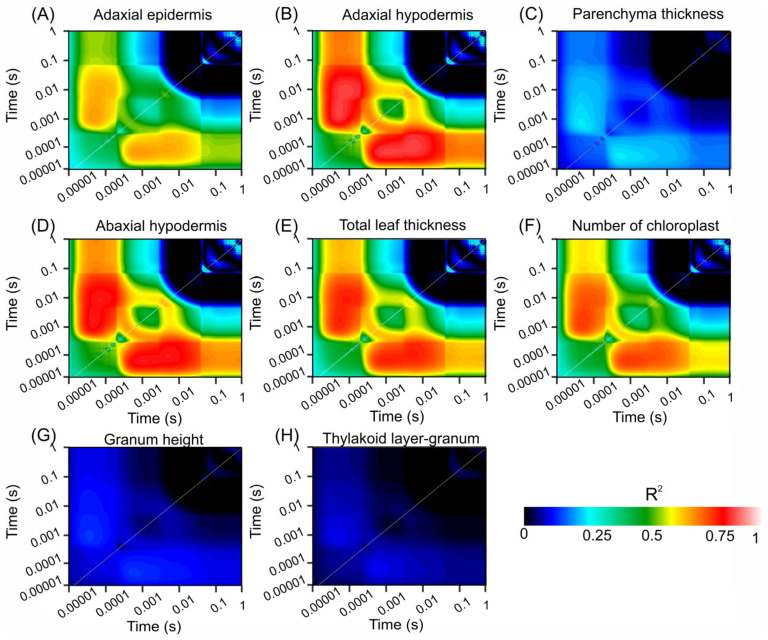
Count plot map of the coefficient of correlation (R^2^). (**A**) Adaxial epidermis. (**B**) Adaxial hypodermis. (**C**) Parenchyma thickness. (**D**) Adaxial hypodermis. (**E**) Total leaf thickness. (**F**) Number of chloroplasts. (**G**) Granum height. (**H**) Thylakoid layer-granum. Dark blue to light red scale indicates increased associations. (*n* = 200).

Figure 5 shows contour plots of the coefficient of determination (R^2^) values from linear regression analyses that explored the relationships between various biophysical compounds and chlorophyll fluorescence (ChlF) over a time span ranging from 0.00001 to 1 s. These plots emphasize the strength of the correlation between fluorescence signals and structural leaf attributes, such as adaxial epidermis, adaxial hypodermis, parenchyma thickness, abaxial hypodermis, total leaf thickness, chloroplast count, granum height, and thylakoid layer granum.

Figure 5A displays the R^2^ values for the adaxial epidermis, which were significantly correlated at the early time points. The transition from dark blue to light red signifies a strong relationship, indicating a rapid response of the adaxial epidermis to fluorescence changes. Similarly, Figure 5B (adaxial hypodermis) and Figure 5D (abaxial hypodermis) show significant early correlations, highlighting the crucial role of these layers in ChlF dynamics.

In contrast, Figure 5C shows lower R^2^ values for parenchyma thickness across all time points, depicted predominantly by blue hues, suggesting a weaker association with the fluorescence parameters. Figure 5E (total leaf thickness) and Figure 5F (chloroplast count) show notable correlations at early and intermediate time points, with higher R^2^ values (green to red transition), indicating their significant roles in ChlF characteristics.

Figure 5G,H show minimal correlations for the granum height and thylakoid layer–granum ratio, respectively, as indicated by the extensive dark blue regions, suggesting a lesser impact on fluorescence within the measured timeframe.

These contour plots visually represent the temporal and quantitative relationships between the chlorophyll fluorescence dynamics and various leaf structural components. The color gradation from dark blue to light red highlights the strongest relationships, providing insight into the most informative moments for ChlF concerning these structural attributes.

Figures S2–S4 present contour plots that illustrate the coefficient of determination (R^2^) values from linear regression analyses, which were used to assess the relationships between the hyperspectral data (reflectance, transmittance, and absorbance) at two wavelengths and various leaf structural attributes. These attributes include adaxial epidermis, adaxial hypodermis, parenchyma thickness, abaxial hypodermis, total leaf thickness, chloroplast count, granum height, and thylakoid layer-to-granum ratio.

Figure S2 displays the R^2^ values for the hyperspectral reflectance data. In Figure S2A, the adaxial epidermis shows strong correlations at specific wavelengths, particularly in the blue and near-infrared (NIR) regions, as indicated by the gradient from dark blue to light red. Similarly, Figure S2B shows significant correlations for the adaxial hypodermis, with high R^2^ values concentrated in the blue- and red-edge regions. Conversely, parenchyma thickness (Figure S2C) generally resulted in lower R^2^ values across most wavelength pairings, implying a weaker relationship with the reflectance data.

Figure S2D highlights the R^2^ values for the abaxial hypodermis, which are strongly correlated at various wavelengths, particularly in the green and NIR regions. Figure S2E,F show notable correlations for total leaf thickness and chloroplast count, respectively, at several wavelengths. Strong correlations for total leaf thickness were observed in the blue and NIR regions, whereas correlations for chloroplast count were more dispersed. Figure S2G,H depict the minimal correlations for the granum height and thylakoid layer-to-granum ratio, respectively, suggesting a lesser impact on the reflectance within the measured spectral range.

Figure S3 shows contour maps of the R^2^ values obtained from the linear regression analysis of the hyperspectral transmittance data. Figure S3A shows strong correlations for the adaxial epidermis at specific wavelengths within the visible (VIS) and NIR spectra. Similarly, Figure S3B shows significant correlations for the adaxial hypodermis, with high R^2^ values in the VIS and NIR spectra. For parenchyma thickness (Figure S3C), lower R^2^ values were observed across various wavelength combinations, indicating a less robust correlation. Figure S3D highlights the significant correlations for the abaxial hypodermis, especially in the green and NIR regions. Figure S3E,F show discernible correlations for the total leaf thickness and chloroplast count, respectively, across multiple wavelengths. Finally, Figure S3G,H present the nominal correlations for the granum height and thylakoid layer-to-granum ratio, respectively.

Figure S4 shows the R^2^ values obtained from the linear regression analysis of the hyperspectral absorbance data. Figure S4A shows significant correlations for the adaxial epidermis at specific wavelengths, particularly in the VIS and NIR regions at approximately 700 and 1600 nm. Figure S4B shows notable correlations for the adaxial hypodermis, with high R^2^ values concentrated at approximately 700 and 1500–1700 nm. Figure S4C shows lower R^2^ values for parenchyma thickness, suggesting a weaker relationship with absorbance data. Figure S4D highlights the strong correlations for the abaxial hypodermis, especially in the green and NIR regions. Figure S4E,F show notable correlations for total leaf thickness and chloroplast count, respectively, at several wavelengths. Finally, Figure S4G,H show minimal correlations for the granum height and thylakoid layer-to-granum ratio, respectively, suggesting a lesser impact on the absorbance within the measured spectral range.

### 3.6. Parameters Predicted by Biophysical Compounds

#### Calibration, Validation and Prediction Models

Table 2 and Table S1 present the statistical metrics from the partial least squares regression (PLSR) models used to predict the biophysical parameters of *Tradescantia* species via various spectrometric methods. These models were assessed based on their calibration and cross-validation performance using fluorescence, reflectance, transmittance, and absorbance measurements.

The calibration model for adaxial epidermal thickness using fluorescence sensors had an R^2^ of 0.92, which remained consistent during cross-validation. The root mean square error (RMSE) values were 6.0 for calibration and 6.3 for cross-validation, with relative prediction deviations (RPDs) of 2.62 and 2.52, respectively, indicating model robustness. For the adaxial hypodermis, the model performed similarly well, with an R^2^ of 0.93 in both calibration and cross-validation, RMSE values of 21.5 and 22.5, and RPD values of 2.79 and 2.68, respectively. The parenchyma thickness predictions exhibited moderate accuracy, with calibration and cross-validation R^2^ values of 0.69 and 0.66, RMSE values of 3.0 and 3.2, and RPD values of 1.38 and 1.34, respectively.

Reflectance measurements for the adaxial epidermis yielded calibration and cross-validation R^2^ values of 0.88 and 0.87, respectively. The RMSE values were 7.4 for calibration and 8.0 for cross-validation, with RPD values of 2.14 and 2.00, respectively. For the adaxial hypodermis, high accuracy was achieved, with R^2^ values of 0.91 for calibration and 0.89 for cross-validation, RMSE values of 25.6 and 27.6, and RPD values of 2.36 and 2.20, respectively. Parenchyma thickness predictions were moderately accurate, with calibration and cross-validation R^2^ values of 0.70 and 0.65, RMSE values of 3.0 and 3.2, and RPD values of 1.39 and 1.32, respectively.

Transmittance measurements for the adaxial epidermis indicated R^2^ values of 0.88 for calibration and 0.87 for cross-validation, with RMSE values of 7.4 and 7.9, and RPD values of 2.14 and 2.01, respectively. For the adaxial hypodermis, high accuracy was demonstrated, with calibration and cross-validation R^2^ values of 0.90 and 0.88, RMSE values of 26.3 and 28.3, and RPD values of 2.29 and 2.15, respectively. The R^2^ values for predicting parenchyma thickness were 0.75 for calibration and 0.70 for cross-validation, with RMSE values of 2.7 and 3.0 and RPD values of 1.52 and 1.40, respectively.

The absorbance measurements for the adaxial epidermis achieved R^2^ values of 0.89 for calibration and 0.88 for cross-validation, with RMSE values of 7.1 and 7.7 and RPD values of 2.23 and 2.07, respectively. The adaxial hypodermis model showed high accuracy, with calibration and cross-validation R^2^ values of 0.92 and 0.90, RMSE values of 23.8 and 26.0, and RPD values of 2.53 and 2.33, respectively. The predictions of parenchyma thickness showed moderate accuracy, with calibration and cross-validation R^2^ values of 0.73 and 0.67, RMSE values of 2.8 and 3.1, and RPD values of 1.47 and 1.35, respectively.

For the prediction parameters of fluorescence, the model for adaxial epidermis thickness demonstrated moderate predictability, with an R^2^ of 0.44 and an RMSEP of 22.7. The adaxial hypodermis showed higher accuracy, with an R^2^ of 0.74 and an RMSEP of 47.1, indicating model robustness. In contrast, parenchyma thickness and abaxial hypodermis had limited predictive accuracy, with R^2^ values of 0.18 and 0.17, respectively. The model for the chloroplast count had a moderate R^2^ of 0.57 and an RMSEP of 3.4, suggesting reasonable reliability, as shown in Table 2.

Reflectance-based models generally produce relatively high R^2^ values. The adaxial epidermis model had an R^2^ of 0.59, whereas the adaxial hypodermis model had an R^2^ of 0.88, indicating better predictability than fluorescence. However, parenchyma thickness showed limited accuracy, with an R^2^ of 0.07. The model for total leaf thickness exhibited a notable R^2^ value of 0.77, demonstrating a strong predictive power.

Transmittance measurements yielded robust R^2^ values for both the adaxial and abaxial hypodermis, at 0.84, with RMSEP values of 34.7 and 20.5, respectively. The model for total leaf thickness also had a high R^2^ value of 0.74, indicating strong predictability.

The absorbance-based models improved the R^2^ values for most parameters. The adaxial epidermis model had an R^2^ of 0.60, whereas the adaxial hypodermis model achieved an R^2^ of 0.89, both of which indicated moderate-to-strong predictive capabilities. The model for total leaf thickness exhibited a strong R^2^ value of 0.78, indicating high reliability.

Overall, the PLSR models displayed varying degrees of predictability across different spectroscopic methods. Fluorescence and reflectance methods generally provided moderate predictability, whereas transmittance and absorbance methods showed greater accuracy for specific parameters. The RMSEP and RPD values indicated the precision and reliability of these models, with higher RPD values suggesting superior predictive capabilities, particularly for structural parameters, such as the adaxial and abaxial hypodermis. The bias values highlighted areas for potential model refinement to enhance the prediction accuracy.

A comparison of the predicted and observed values in the scatter plots revealed varying prediction accuracies across the different spectroscopic methods.

For the fluorescence sensor (Figure S5), strong correlations were observed for adaxial and abaxial hypodermis thickness (R^2^ = 0.94) and total leaf thickness (R^2^ = 0.87), indicating a high predictive power. However, parameters such as parenchyma thickness (R^2^ = −0.01) and thylakoid layer–granum ratio (R^2^ = 0.05) exhibited weak correlations, suggesting poor predictability.

The reflectance data (Figure S6) showed robust R^2^ values for the adaxial hypodermis thickness (0.8) and abaxial hypodermis thickness (0.91), indicating strong predictive relationships. Similar to fluorescence, parenchyma thickness (R^2^ = 0.18) and the thylakoid layer–granum ratio (R^2^ = 0.05) were predicted less accurately.

The transmittance data (Figure S7) displayed moderate to strong correlations for most parameters. The thicknesses of the adaxial (R^2^ = 0.87) and abaxial hypodermis (R^2^ = 0.92) were highly predictable. However, parenchyma thickness (R^2^ = 0.16) and thylakoid layer–granum ratio (R^2^ = 0.07) showed weak predictability.

The absorbance data (Figure S8) demonstrated moderate predictive accuracy, with strong correlations between adaxial hypodermis thickness (R^2^ = 0.94) and total leaf thickness (R^2^ = 0.8). Parenchyma thickness (R^2^ = −0.04) and the thylakoid layer–granum ratio (R^2^ = 0.06) were poorly correlated, indicating limitations in the use of absorbance for these measurements.

In summary, the analysis revealed that parameters such as hypodermis and total leaf thickness, particularly fluorescence and reflectance, were consistently well predicted across all methods. Conversely, parameters such as parenchyma thickness and the thylakoid layer–granum ratio exhibited low predictability, highlighting areas where model refinement was needed. A higher green color intensity in the absorbance data plots indicates stronger correlations for specific parameters, suggesting that absorbance could be a more reliable predictor of biophysical compounds.

## 4. Discussion

Considerable progress has been made by integrating hyperspectral sensors to predict the biophysical parameters of plants. Chlorophyll fluorescence and hyperspectral sensors collect extensive data, facilitating detailed and precise predictions that are closely aligned with the physiological and biophysical functions. The incorporation of hyperspectral vegetation indices (HVIs), along with diverse analytical tools, has led to significant enhancements in monitoring and forecasting various structural and ultrastructural compounds [60,61].

Our fluorescence, reflectance, transmittance, and absorbance measurements demonstrated varying degrees of predictive accuracy. For example, chlorophyll a fluorescence was highly effective in predicting parameters such as adaxial and abaxial hypodermal thickness (R^2^ = 0.94) and total leaf thickness (R^2^ = 0.87). Similarly, reflectance data showed strong predictive power for adaxial hypodermis thickness (R^2^ = 0.80) and abaxial hypodermis thickness (R^2^ = 0.91). Transmittance readings also yielded robust predictions for adaxial (R^2^ = 0.87) and abaxial hypodermal thickness (R^2^ = 0.92). Absorbance measurements provided high predictive accuracy for adaxial hypodermis thickness (R^2^ = 0.94) and total leaf thickness (R^2^ = 0.80). Other studies that sought to relate different spectral curves using similar approaches reported comparable values [24,25,45]. The findings presented here align with the key advancements our research group has made from 2017 to 2024, emphasizing the importance of analyzing spectral curves using reflectance, transmittance, and absorbance [10,11,29,30,31,59,62,63,64,65,66]. These distinct modes of spectral acquisition capture the varied interactions between the electromagnetic spectrum and leaf optical properties, offering a more accurate representation of the leaf biophysical processes. Additionally, our recent integration of fluorescence sensors with infrared gas analysis (IRGA) has improved the analysis of not only biochemical aspects but also the structural and ultrastructural changes across different leaf types, enhancing our overall understanding of leaf biophysics and plant function [10,11,29,30,31,59,62,63,64,65,66].

However, some parameters, such as parenchyma thickness and the thylakoid layer–granum ratio consistently exhibited lower predictability across all sensor types. This suggests that the current models may not fully capture the complexity of these structures, potentially owing to limitations in sensor sensitivity or spectral resolution for these specific measurements. The variability in tissue composition, structural heterogeneity, and influence of overlapping biophysical factors could also contribute to reduced accuracy. These findings indicate that further model refinement, perhaps through the integration of additional sensor types or advanced analytical techniques, is necessary to improve the predictive accuracy of these parameters. These limitations highlight the need for a more tailored approach to measuring traits that exhibit subtle optical responses [5,11,29].

The predictive models employed in this study highlight the importance of integrating multiple sensors. The high R^2^ values for the key parameters validate the reliability of these models in estimating various pigment concentrations, establishing a basis for future research and practical applications in precision agriculture. The strong performance of our models across different spectroscopic methods confirmed their potential for improving plant health assessments and environmental monitoring.

This is consistent with existing studies that highlight the importance of non-imaging hyperspectral sensors in remote sensing and improve sustainability on the basis of many factors, such as photochemical, biochemical, and metabolic processes, as well as the mechanisms of plant photosynthetic reactions and their interactions with biotic and abiotic stresses on the physiological states of plants and green chemistry. The interactions between light and plant tissues, such as absorption, reflection, and transmission, provide crucial insights into physiological processes. In other studies, the interactions for the precise prediction of plant compounds enhanced the field of remote sensing and facilitated the use of optical spectroscopy for further sample characterization [3,17,19].

Hyperspectral vegetation indices (HVIs) are particularly effective for assessing a broad spectrum of physiological, biochemical, and structural parameters. These indices are vital for monitoring environmental stresses such as water and nitrogen deficiencies. For example, narrowband HVIs are highly sensitive to fluctuations in water and nitrogen levels, allowing for detailed evaluation of crop responses under different irrigation and fertilization conditions [51,67].

Combining HVIs with hyperspectral measurements increases the precision of agricultural interventions, optimizes resource utilization, and enhances crop management strategies. The clear spectral differences observed in the reflectance, transmittance, and absorbance curves of *T. spathacea* and *T. pallida* further underscore the utility of these methods for species differentiation and physiological and green chemistry evaluations [11].

### 4.1. Hyperspectral and Principal Component Analyses of Reflectance, Transmittance, and Absorbance

Hyperspectral sensors reveal detailed spectral data that facilitate comprehensive analysis of plant biochemical and biophysical properties. Reflectance measurements provide essential insights into both the surface and internal structures of leaves, revealing variations in the chlorophyll content and potential cellular changes. This finding is consistent with previous research, suggesting that changes in reflectance can indicate physiological alterations in plant tissues. Similarly, transmittance data are critical for understanding the light penetration and distribution within a leaf, offering insights into its structural and functional attributes.

Given the complexity of hyperspectral datasets, advanced data-reduction techniques, such as principal component analysis (PCA), are indispensable. In this study, PCA was successfully applied to identify the key spectral regions associated with variations in chlorophyll a fluorescence sensors. This is important for developing robust sensors capable of real-time monitoring of multiple biophysical parameters associated with complex environments, which significantly enhances the precision of sensor-fusion methods for measurements. The predictive performance of these hyperspectral measurements was rigorously evaluated with a focus on the coefficients of variation and value ranges to ensure data reliability across the electromagnetic spectrum. Our findings demonstrate that appropriately applied passive sensor techniques provide detailed insights into plant health and photosynthetic efficiency, which are crucial for accurate plant phenotyping and leaf tissue alterations [24,68,69].

Our study also investigated the integration of various sensor types, including hyperspectral and chlorophyll fluorescence sensors, to enhance the predictive modeling capabilities [12,70]. This combination broadens the applicability of the data, improves the accuracy of the predictions, and monitors diverse plant health parameters. Therefore, hyperspectral non-imaging techniques have the potential to revolutionize plant physiological research and agricultural practices. By providing a deeper molecular-level understanding of plant dynamics, these methods offer rapid and precise insights into plant health and phenotyping, which are essential for improving agricultural efficiency and accuracy [71].

Moreover, our results align with recent advancements in hyperspectral technology that emphasize the importance of integrating reflectance, transmittance, and absorbance data for a more comprehensive analysis of plant health, which aligns with tissue, cell, and organelle analyses. Advanced algorithms and machine learning techniques are now being used to further increase the predictive power and accuracy of hyperspectral data, making them vital tools in modern agricultural practice [17,28,46].

Additionally, recent research has demonstrated the potential of hyperspectral non-imaging to detect early signs of plant stress before they become visually apparent [11,17,28,29,46]. This early detection capability is crucial for implementing timely interventions to mitigate stress factors such as nutrient deficiencies, pest infestations, or water stress. By identifying spectral signatures associated with specific stress responses, hyperspectral imaging can facilitate the development of targeted treatment strategies, thereby increasing crop resilience and yield [3,24,44,72].

Furthermore, the integration of hyperspectral data with other remote-sensing technologies, such as LiDAR and thermal imaging, presents a promising avenue for a more holistic understanding of plant health. LiDAR can provide detailed three-dimensional (3D) structural information, whereas thermal imaging offers insights into plant water status and temperature regulation. Combining these datasets with hyperspectral information allows for a multifaceted analysis of plant physiology, leading to more precise and comprehensive monitoring systems [1,40,73].

Finally, the scalability of hyperspectral non-imaging technology is crucial for its application in agriculture. Advances in sensor miniaturization and cost reduction have made these sensors more accessible for both small- and large-scale farming. In this study, combining two sensors to measure reflectance and transmittance and then calculating absorbance, along with fluorescence measurements, provided a more comprehensive analysis of leaf optical properties. This approach enhances the ability to detect structural and biochemical changes in plants and offers deeper insights than the use of a single sensor alone. In this sense, the development of portable hyperspectral devices and their integration with UAVs enables high-resolution regular monitoring over large areas. This supports precision agriculture by improving resource efficiency and reducing environmental impacts. Similar studies have highlighted that the use of multiple sensors not only improves measurement accuracy but also expands the range of observable traits, providing more detailed information for advancing agricultural research [11,17,28,29,46].

### 4.2. Predictive Modeling-Based Reflectance, Transmittance, and Absorbance

Merging hyperspectral data with partial least squares (PLS) modeling has emerged as a transformative approach in plant science, greatly improving our ability to predict and understand intricate physiological traits. Integrating measurements of reflectance, transmittance, and absorbance with advanced multivariate algorithms, such as PLS, highlights the essential role of these parameters in reflecting photosynthetic efficiency and electron transport chain functionality. This integration significantly enhances the precision of the predictive models [74,75].

Variable Importance in Projection (VIP), many machine and deep learning algorithm scores, and hyperspectral vegetation indices (HVIs) are critical for identifying the most informative wavelengths in hyperspectral datasets, thus improving the accuracy of predictive models and allowing for a detailed understanding of plant characteristics. These methods underscore the importance of identifying spectral regions that are rich in information and are closely related to the physiological state of plants [63,76,77].

Principal component analysis (PCA) is employed to handle the complexity of large-scale hyperspectral data by pinpointing key spectral regions associated with variations in chlorophyll a fluorescence parameters. These spectral signatures are crucial for creating remote sensing tools that can noninvasively evaluate plant properties such as chlorophyll and nitrogen contents [78,79,80].

The predictive models derived from this integrated approach enable real-time analysis, thereby reinforcing the efficacy of remote sensing for extensive monitoring. This aligns with the current views on the transformative potential of these technologies for global monitoring and productivity management. Real-time monitoring is crucial for informed decision-making in agriculture, forestry, and other sectors that rely on remote sensing, enhancing productivity, and sustainability [2,81].

Importantly, although significant progress has been made in applying hyperspectral and PLS models, further research is necessary to extend these techniques to diverse plant species and environmental conditions. Whole-spectrum models, such as PLSR, LDA, and SVR, have shown great efficacy in classifying and predicting leaf properties. These models provide valuable insights for interpreting chlorophyll a fluorescence data, which is crucial for JIP test evaluations. By utilizing the full spectrum of available data, these models ensure a thorough analysis of complex leaf attributes, including the interactions between leaf optical properties and the intricate chemistry of molecules in variegated leaves [45,82].

In conclusion, a variety of spectroscopic methods, including those with and without non-imaging (proximal sensor) functions, have been extensively employed to decipher details about photochemical, biochemical, biophysical, and metabolic activities, as well as to understand how plant photosynthesis reacts to and interacts with living organisms and environmental stressors, influencing the physiological conditions of plants by accurate responses.

## 5. Conclusions

The use of hyperspectral and chlorophyll detection technologies highlights their efficacy in assessing biochemical elements in *Tradescantia* species, thereby offering a thorough understanding of plant health and structural anomalies. These methodologies demonstrated high accuracy and reliability through rigorous statistical analyses, confirming their suitability for sensor technology and chemometric studies. Identifying critical spectral zones spanning from ultraviolet—blue to shortwave infrared is essential for noninvasive biochemical measurements and cellular structure detection. The use of hyperspectral vegetation indices (HVIs) significantly improves the performance of partial least squares (PLS) regression models. The precision of these models underscores the importance of stationary hyperspectral and fluorescence probes for analyzing diverse foliage. The insights from this research establish a crucial framework for integrating various standoff sensors and hyperspectral imaging methods in botanical studies. Future research should expand these techniques to encompass a wider variety of plant species and environmental conditions to enhance the robustness and applicability of these models. Advancing these approaches will confirm the pivotal role of hyperspectral sensing technologies in agricultural surveillance and management.

## Figures and Tables

**Table 1:** Descriptive statistics of the biophysical parameters. The data included the mean, median, minimum, maximum, and coefficient of variation for each parameter. (*n* = 200).

Parameter	Count (n)	Mean	Median	Minimum	Maximum	CV (%)
Adaxial epidermis (μm)	200	102.7	88.0	61	162	23.94
Adaxial hypodermis (μm)	200	323.4	325.7	212	452	25.79
Parenchyma thickness (μm)	200	252.6	251.3	224	296	3.80
Abaxial hypodermis (μm)	200	170.5	164.7	117	246	25.53
Total leaf thickness (μm)	200	849.1	820.5	735	978	7.02
Number of chloroplast	200	21.2	20.5	14	32	22.23
Granum height (nm)	200	522.5	531.9	75	1000	37.29
Thylakoid layer-granum	200	25.2	26.0	4.0	53.0	33.80

**Table 2:** The performance of partial least squares regression (PLSR) models that use various spectroscopic techniques to estimate biophysical compound concentrations in *Tradescantia* species. The evaluation metrics included the correlation coefficient (r), R-squared (R^2^), slope, offset, standard error of prediction (SEP), root mean squared error of prediction (RMSEP), ratio of performance to deviation (RPD), and bias, offering a detailed analysis for each sensor. (*n* = 50).

Sensors	Parameter	Maximum Factor PLS	Predicted							
			r	R^2^	Slope	Offset	SEP	RMSEP	RPD	Bias
**Fluorescence**	Adaxial epidermis (μm)	5	0.66	0.44	0.54	45.80	22.80	22.70	1.33	2.14
	Adaxial hypodermis (μm)	5	0.86	0.74	0.95	17.80	47.50	47.10	1.95	2.06
	Parenchyma thickness (μm)	4	0.42	0.18	0.14	219.01	14.00	14.20	1.10	2.92
	Abaxial hypodermis (μm)	5	0.42	0.17	1.30	152.80	43.70	88.80	1.10	77.53
	Total leaf thickness (μm)	5	0.77	0.59	0.77	203.70	43.40	43.60	1.56	7.28
	Number of chloroplast	5	0.76	0.57	0.75	5.50	3.40	3.40	1.53	0.36
	Granum height (nm)	2	0.20	0.04	0.09	522.20	204.90	246.00	1.02	138.62
	Thylakoid layer-granum	2	0.10	0.01	0.04	25.30	11.00	11.40	1.01	3.30
**Reflectance**	Adaxial epidermis (μm)	7	0.77	0.59	0.48	51.90	19.60	19.50	1.57	2.40
	Adaxial hypodermis (μm)	6	0.94	0.88	0.81	62.00	29.10	28.90	2.91	1.80
	Parenchyma thickness (μm)	6	0.26	0.07	0.07	236.50	14.80	15.00	1.04	3.10
	Abaxial hypodermis (μm)	7	0.94	0.88	0.79	39.20	15.80	16.30	2.84	4.50
	Total leaf thickness (μm)	7	0.88	0.77	0.68	274.30	31.00	31.50	2.10	6.90
	Number of chloroplast	7	0.88	0.77	0.67	7.20	2.50	2.40	2.09	0.40
	Granum height (nm)	7	0.11	0.01	0.06	539.10	217.10	256.80	1.01	140.00
	Thylakoid layer-granum	7	0.03	0.00	0.01	25.60	11.60	11.90	1.00	3.10
**Transmittance**	Adaxial epidermis (μm)	7	0.76	0.57	0.55	41.20	19.50	20.10	1.53	5.30
	Adaxial hypodermis (μm)	7	0.92	0.84	0.91	18.60	33.70	34.70	2.51	9.30
	Parenchyma thickness (μm)	6	0.25	0.06	0.08	234.30	15.00	15.30	1.03	3.80
	Abaxial hypodermis (μm)	7	0.92	0.84	0.91	26.70	17.50	20.50	2.52	10.80
	Total leaf thickness (μm)	7	0.86	0.74	0.77	196.50	32.30	32.00	1.96	0.10
	Number of chloroplast	6	0.82	0.68	0.77	4.70	2.80	2.80	1.76	0.20
	Granum height (nm)	7	0.14	0.02	0.07	503.30	210.80	236.10	1.01	109.60
	Thylakoid layer-granum	6	0.13	0.02	0.04	24.20	10.90	11.10	1.01	2.40
**Absorbance**	Adaxial epidermis (μm)	7	0.78	0.60	0.56	42.20	18.90	19.00	1.59	2.90
	Adaxial hypodermis (μm)	7	0.94	0.89	0.95	14.90	27.90	27.70	3.03	0.50
	Parenchyma thickness (μm)	7	0.25	0.06	0.08	235.00	14.90	15.20	1.03	3.40
	Abaxial hypodermis (μm)	7	0.94	0.89	0.93	17.10	14.80	15.90	2.96	6.10
	Total leaf thickness (μm)	7	0.88	0.78	0.80	174.90	29.20	30.30	2.12	6.00
	Number of chloroplast	7	0.88	0.77	0.82	4.30	2.40	2.40	2.09	0.40
	Granum height (nm)	7	0.02	0.00	0.01	556.80	230.70	267.60	1.00	138.90
	Thylakoid layer-granum	6	0.02	0.00	0.01	26.10	12.10	12.40	1.00	3.10

## Data Availability

The original contributions presented in the study are included in the article/Supplementary Material, and further inquiries can be directed to the corresponding author.

## Supplementary Materials

The following supporting information can be downloaded at: https://www.mdpi.com/article/10.3390/s24196490/s1, Figure S1: Spectral leaf reflectance, transmittance, and absorbance profiles from 350 to 2500 nm. (A) Chlorophyll a fluorescence kinetics. (B) Reflectance spectra. (C) Transmittance spectra. (D) Absorbance spectra. In these plots, *T. spathacea* is shown using red–pink lines, while *T. pallida* is represented by green lines. Accuracy (Acc) and Kappa coefficient (K) are noted. (*n* = 100); Figure S2: Reflectance by count plot map of the coefficient of correlation (R^2^). (A) Adaxial epidermis. (B) Adaxial hypodermis. (C) Parenchyma thickness. (D) Adaxial hypodermis. (E) Total leaf thickness. (F) Number of chloroplasts. (G) Granum height. (H) Thylakoid layer-granum. Dark blue to light red indicates increased associations. (*n* = 200); Figure S3: Transmittance by count plot map of the coefficient of correlation (R^2^). (A) Adaxial epidermis. (B) Adaxial hypodermis. (C) Parenchyma thickness. (D) Adaxial hypodermis. (E) Total leaf thickness. (F) Number of chloroplasts. (G) Granum height. (H) Thylakoid layer-granum. Dark blue to light red indicates increased associations. (*n* = 200); Figure S4: Absorbance count plot map of the coefficient of correlation (R^2^). (A) Adaxial epidermis. (B) Adaxial hypodermis. (C) Parenchyma thickness. (D) Adaxial hypodermis. (E) Total leaf thick-ness. (F) Number of chloroplasts. (G) Granum height. (H) Thylakoid layer-granum. Dark blue to light red indicates increased associations. (*n* = 200); Figure S5: Observed vs predicted data estimated by partial least squares regression (PLSR) with hyperspectral ChlF data. (*n* = 50); Figure S6: The observed vs. predicted data were estimated using partial least squares regression (PLSR) with hyperspectral reflectance data. (*n* = 50); Figure S7: The measured data were compared to the data calculated via partial least squares regression (PLSR) with hyperspectral transmittance data. (*n* = 50); Figure S8: Observed vs. predicted data estimated by partial least squares regression (PLSR) of hyperspectral absorbance data. (*n* = 50); Table S1: The performance of partial least squares regression (PLSR) models that use various spectroscopic techniques to estimate biophysical compound concentrations in *Tradescantia* species. The evaluation metrics included the correlation coefficient (r), R-squared (R²), slope, offset, standard error of prediction (SEP), root mean squared error of prediction (RMSEP), ratio of performance to deviation (RPD), and bias, offering a detailed analysis for each sensor. Statistical performance of the PLSR model in predicting phases. Statistical metrics from the PLSR model in the calibration and cross-validation phases. (*n* = 150).
