# Supplementary material for: Fluorescence and Hyperspectral Sensors for Nondestructive Analysis and Prediction of Biophysical Compounds in the Green and Purple Leaves of Tradescantia Plants
Source: Sensors (Basel). 2024 Oct 9;24(19):6490. doi: 10.3390/s24196490 (PMC11479283; doi:10.3390/s24196490)
Supplement: Supplementary file 1 [file sensors-24-06490-s001.zip › sensors-3218587-supplementary.pdf]

sensors

Supplementary file

Fluorescence and Hyperspectral Sensors for Nondestructive Analysis and Prediction of Biophysical Compounds in the Green and Purple Leaves of *Tradescantia* Plants

Renan Falcioni [1,*], Roney Berti de Oliveira [1], Marcelo Luiz Chicati [1], Werner Camargos Antunes [1], José Alexandre M. Demattê [2] and Marcos Rafael Nanni [1]

[1] Department of Agronomy, State University of Maringá, Av. Colombo, 5790, Maringá 87020-900, Paraná, Brazil; rboliveira@uem.br (R.B.d.O.); mlchicati@uem.br (M.L.C.); wcantunes@uem.br (W.C.A.); mrnanni@uem.br (M.R.N.)
[2] Department of Soil Science, Luiz de Queiroz College of Agriculture, University of São Paulo, Av. Pádua Dias, 11, Piracicaba 13418-260, São Paulo, Brazil; jamdemat@usp.br
* Correspondence: renanfalcioni@gmail.com or rfalcioni2@uem.br; Tel.: +55-4430118940

Table S1. The performance of partial least squares regression (PLSR) models that use various spectroscopic techniques to estimate biophysical compound concentrations in *Tradescantia* species. The evaluation metrics included the correlation coefficient (r), R-squared (R^2), slope, offset, standard error of prediction (SEP), root mean squared error of prediction (RMSEP), ratio of performance to deviation (RPD), and bias, offering a detailed analysis for each sensor. Statistical performance of the PLSR model in predicting phases. Statistical metrics from the PLSR model in the calibration and cross-validation phases. (n = 150).

Sensors	Parameter	Maximum Factor PLS	Calibration				Cross-Validation			
			R^2	Offset	RMSE	RPD	R^2	Offset	RMSE	RPD
Fluorescence	Adaxial epidermis (µm)	2	0.92	7.7	6.0	2.62	0.92	7.9	6.3	2.52
	Adaxial hypodermis (µm)	2	0.93	21.6	21.5	2.79	0.93	21.8	22.5	2.68
	Parenchyma thickness (µm)	2	0.69	79.2	3.0	1.38	0.66	81.8	3.2	1.34
	Abaxial hypodermis (µm)	2	0.93	11.7	11.4	2.75	0.93	11.9	11.9	2.64
	Total leaf thickness (µm)	2	0.93	62.1	15.1	2.67	0.92	63.5	16.1	2.56
	Number of chloroplast	2	0.88	2.7	1.7	2.09	0.87	2.7	1.7	2.00
	Granum height (nm)	2	0.22	439.8	154.0	1.03	0.19	448.1	157.1	1.02
	Thylakoid layer-granum	2	0.21	20.8	6.3	1.02	0.17	21.2	6.5	1.02
Reflectance	Adaxial epidermis (µm)	5	0.88	11.9	7.4	2.14	0.87	12.5	8.0	2.00
	Adaxial hypodermis (µm)	4	0.91	30.7	25.6	2.36	0.89	32.4	27.6	2.20
	Parenchyma thickness (µm)	6	0.70	76.9	3.0	1.39	0.65	80.9	3.2	1.32
	Abaxial hypodermis (µm)	4	0.91	16.2	13.4	2.35	0.89	17.2	14.4	2.19
	Total leaf thickness (µm)	5	0.89	91.8	18.8	2.21	0.87	97.7	20.3	2.06
	Number of chloroplast	6	0.85	3.2	1.8	1.89	0.83	3.4	1.9	1.79
	Granum height (nm)	5	0.37	353.6	138.1	1.08	0.31	371.7	144.9	1.05
	Thylakoid layer-granum	7	0.38	16.3	5.6	1.08	0.30	17.3	6.0	1.05
Transmittance	Adaxial epidermis (µm)	7	0.88	11.8	7.4	2.14	0.87	12.5	7.9	2.01
	Adaxial hypodermis (µm)	7	0.90	32.3	26.3	2.29	0.88	33.7	28.3	2.15
	Parenchyma thickness (µm)	7	0.75	62.2	2.7	1.52	0.70	68.3	3.0	1.40
	Abaxial hypodermis (µm)	7	0.91	15.8	13.2	2.37	0.89	16.3	14.3	2.20
	Total leaf thickness (µm)	7	0.88	102.5	19.9	2.10	0.86	107.7	21.3	1.98
	Number of chloroplast	7	0.85	3.3	1.8	1.88	0.83	3.5	1.9	1.77
	Granum height (nm)	6	0.50	282.8	123.5	1.15	0.43	291.1	131.3	1.11
	Thylakoid layer-granum	7	0.47	13.8	5.2	1.14	0.40	14.1	5.5	1.09
Absorbance	Adaxial epidermis (µm)	7	0.89	10.8	7.1	2.23	0.88	10.9	7.7	2.07
	Adaxial hypodermis (µm)	6	0.92	26.5	23.8	2.53	0.90	26.6	26.0	2.33
	Parenchyma thickness (µm)	7	0.73	67.2	2.8	1.47	0.67	71.5	3.1	1.35
	Abaxial hypodermis (µm)	7	0.92	13.6	12.3	2.56	0.90	13.8	13.4	2.34
	Total leaf thickness (µm)	7	0.90	84.61	18.1	2.30	0.88	86.4	19.5	2.14
	Number of chloroplast	7	0.86	3.0	1.7	1.95	0.84	3.1	1.9	1.83
	Granum height (nm)	7	0.38	348.8	137.2	1.08	0.32	364.6	144.0	1.06
	Thylakoid layer-granum	7	0.41	15.41	5.5	1.10	0.31	16.5	5.9	1.05

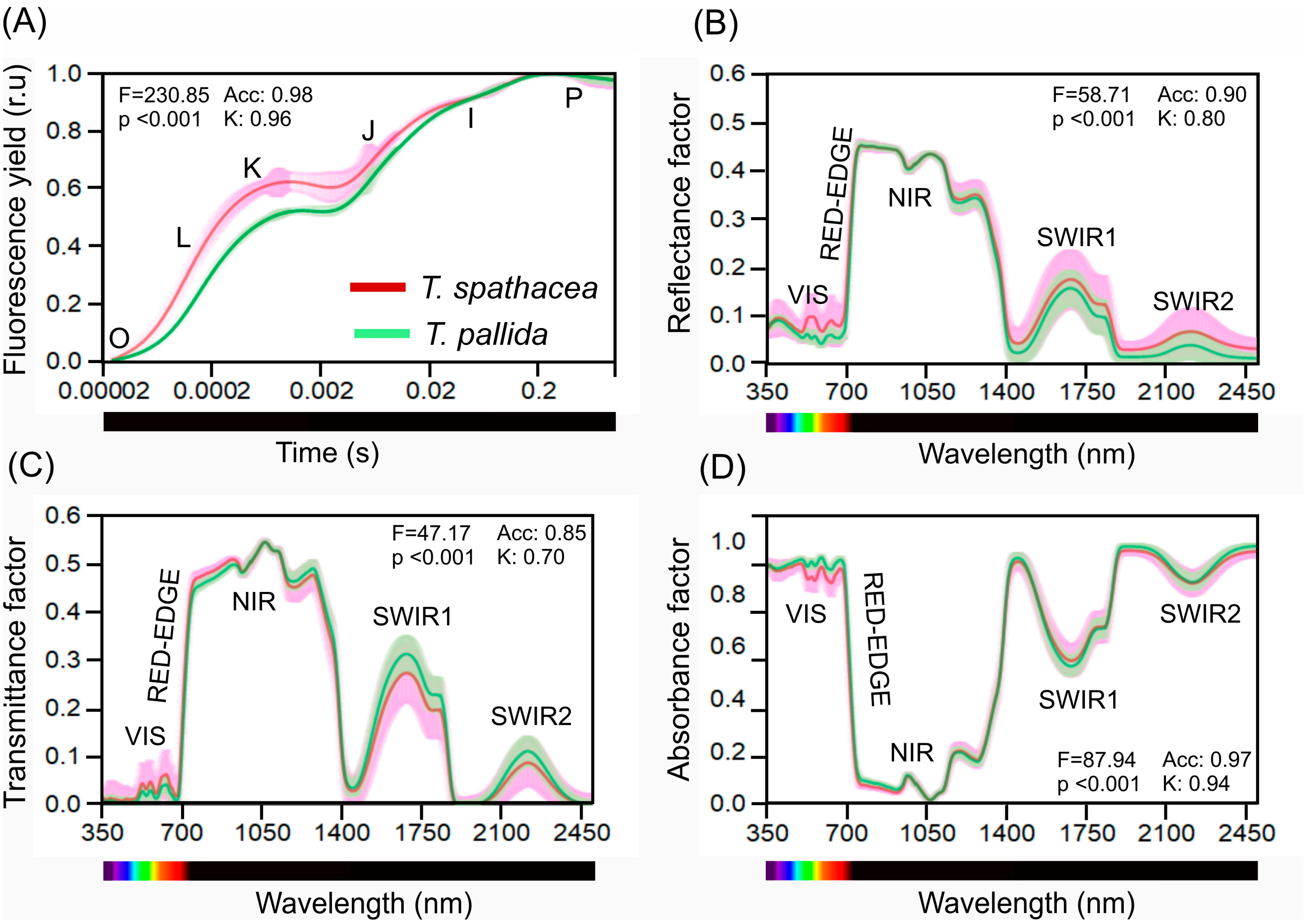

Figure S1. Spectral leaf reflectance, transmittance, and absorbance profiles from 350 to 2500 nm. (**A**) Chlorophyll a fluorescence kinetics. (**B**) Reflectance spectra. (**C**) Transmittance spectra. (**D**) Absorbance spectra. In these plots, *T. spathacea* is shown using red–pink lines, while *T. pallida* is represented by green lines. Accuracy (Acc) and Kappa coefficient (K) are noted. (n = 100).

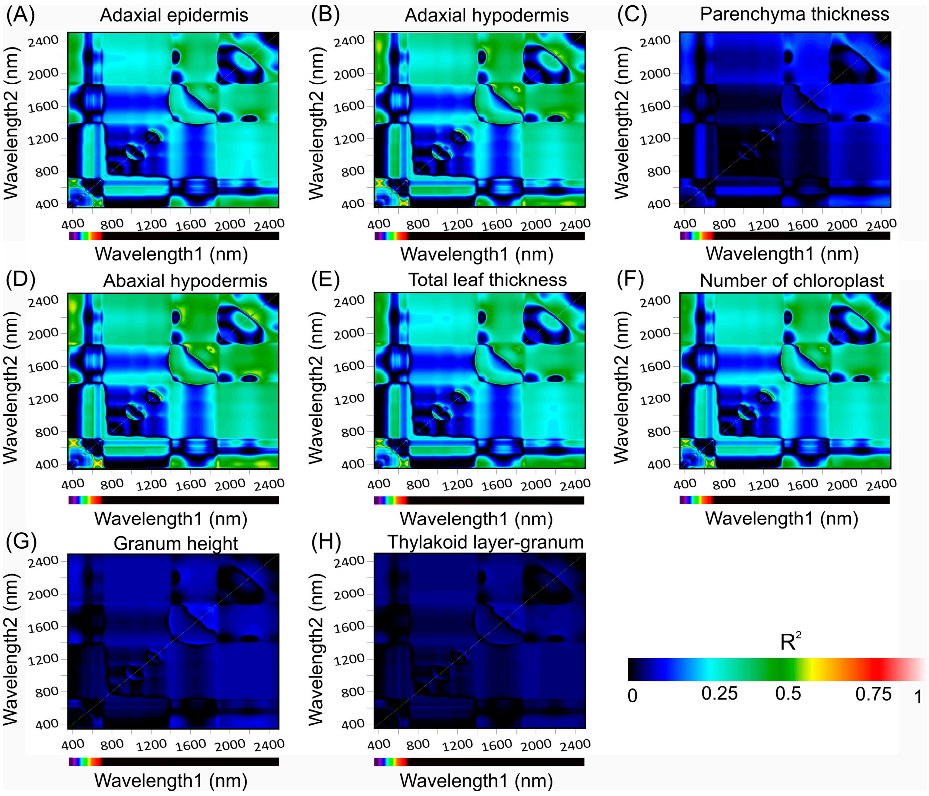

Figure S2. Reflectance by count plot map of the coefficient of correlation (R^2). (**A**) Adaxial epidermis. (**B**) Adaxial hypodermis. (**C**) Parenchyma thickness. (**D**) Adaxial hypodermis. (**E**) Total leaf thickness. (**F**) Number of chloroplasts. (**G**) Granum height. (**H**) Thylakoid layer-granum. Dark blue to light red indicates increased associations. (n = 200).

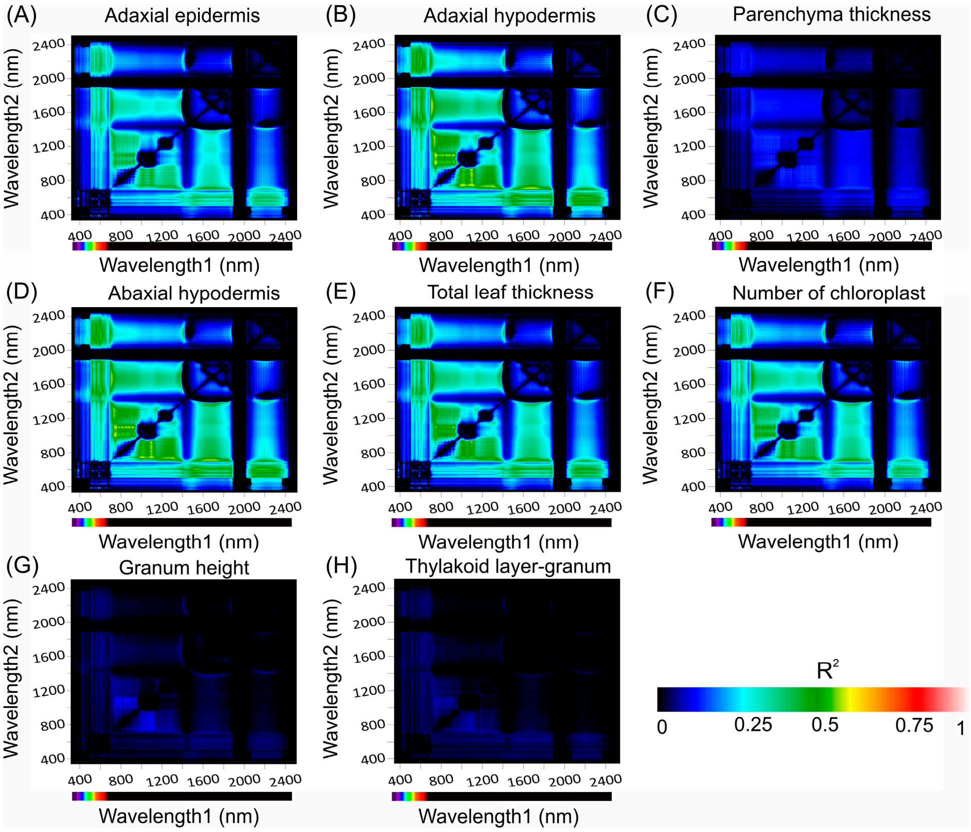

Figure S3. Transmittance by count plot map of the coefficient of correlation (R^2). (**A**) Adaxial epidermis. (**B**) Adaxial hypodermis. (**C**) Parenchyma thickness. (**D**) Adaxial hypodermis. (**E**) Total leaf thickness. (**F**) Number of chloroplasts. (**G**) Granum height. (**H**) Thylakoid layer-granum. Dark blue to light red indicates increased associations. (n = 200).

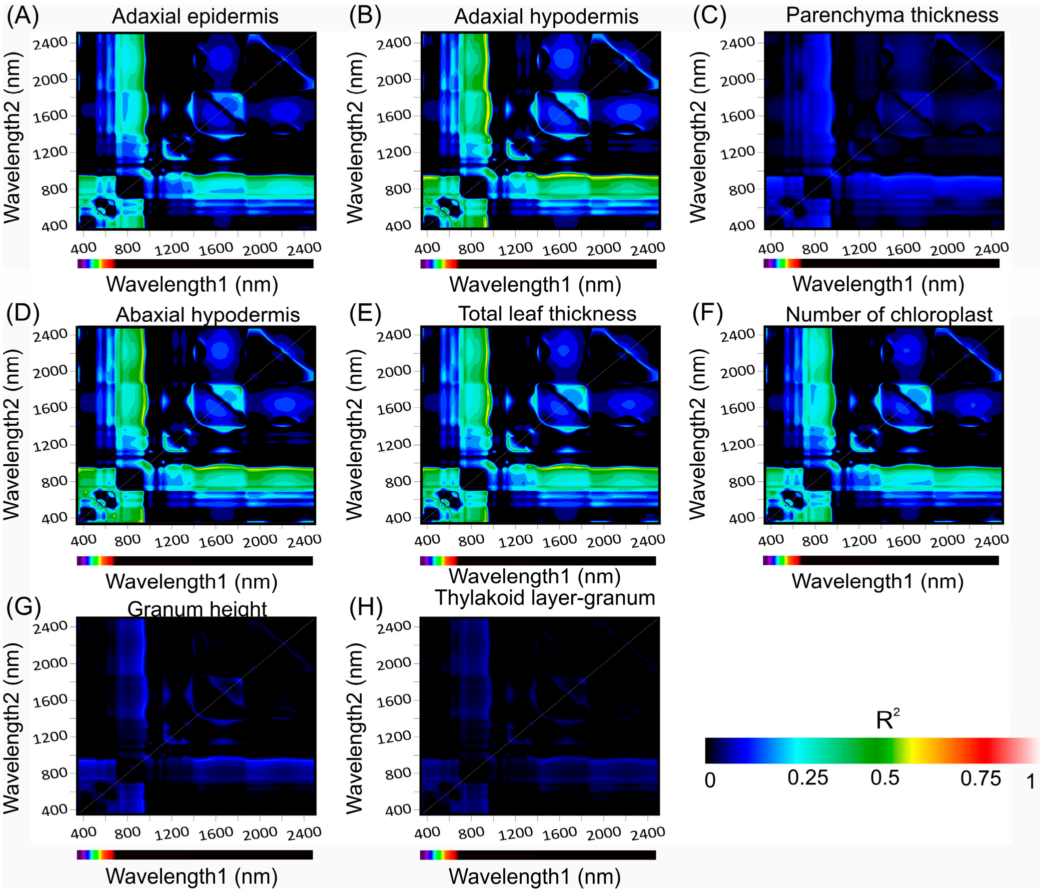

Figure S4. Absorbance count plot map of the coefficient of correlation (R^2). (**A**) Adaxial epidermis. (**B**) Adaxial hypodermis. (**C**) Parenchyma thickness. (**D**) Adaxial hypodermis. (**E**) Total leaf thickness. (**F**) Number of chloroplasts. (**G**) Granum height. (**H**) Thylakoid layer-granum. Dark blue to light red indicates increased associations. (n = 200).

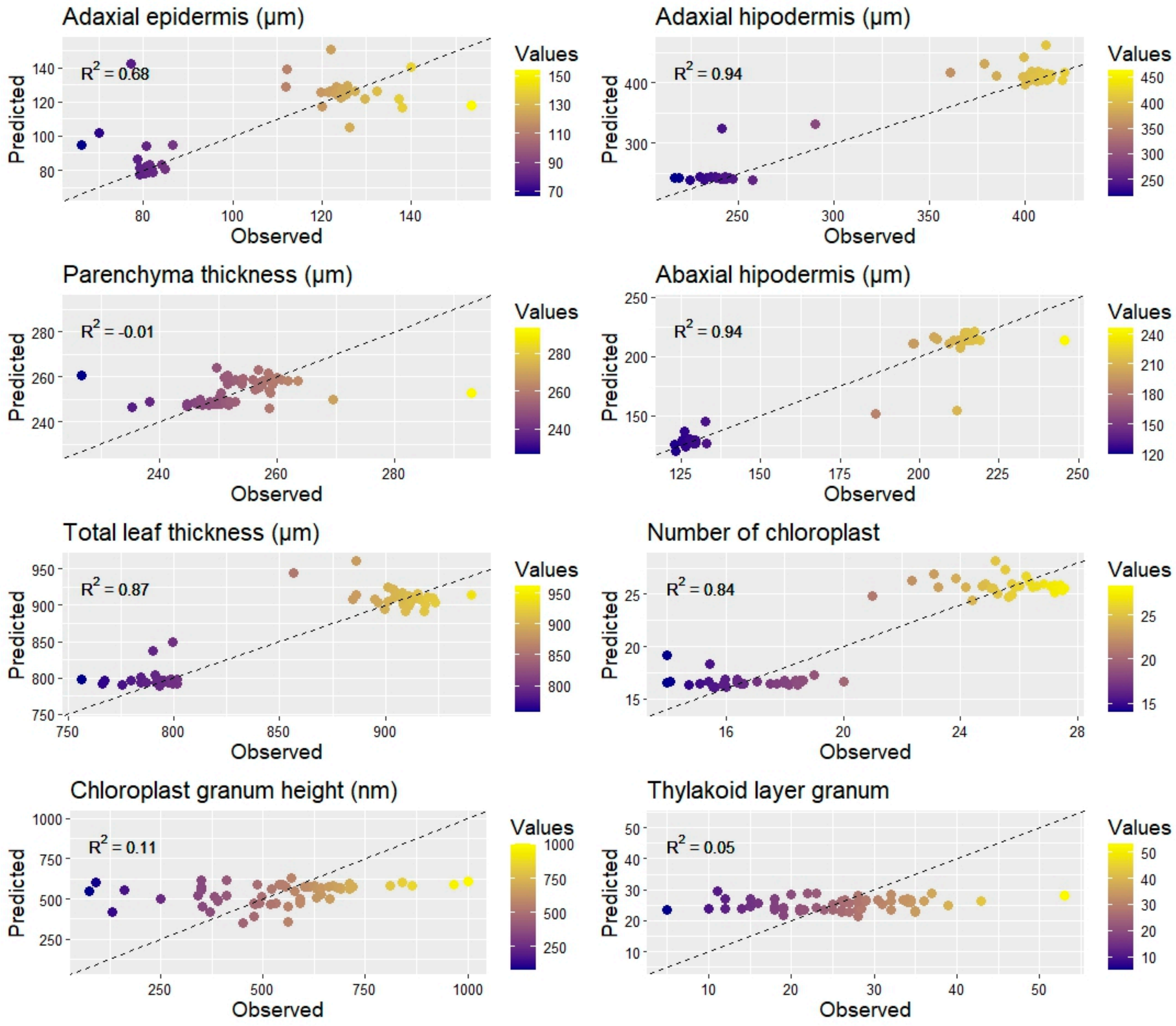

Figure S5. Observed vs. predicted data estimated by partial least squares regression (PLSR) with hyperspectral ChlF data. ($n = 50$).

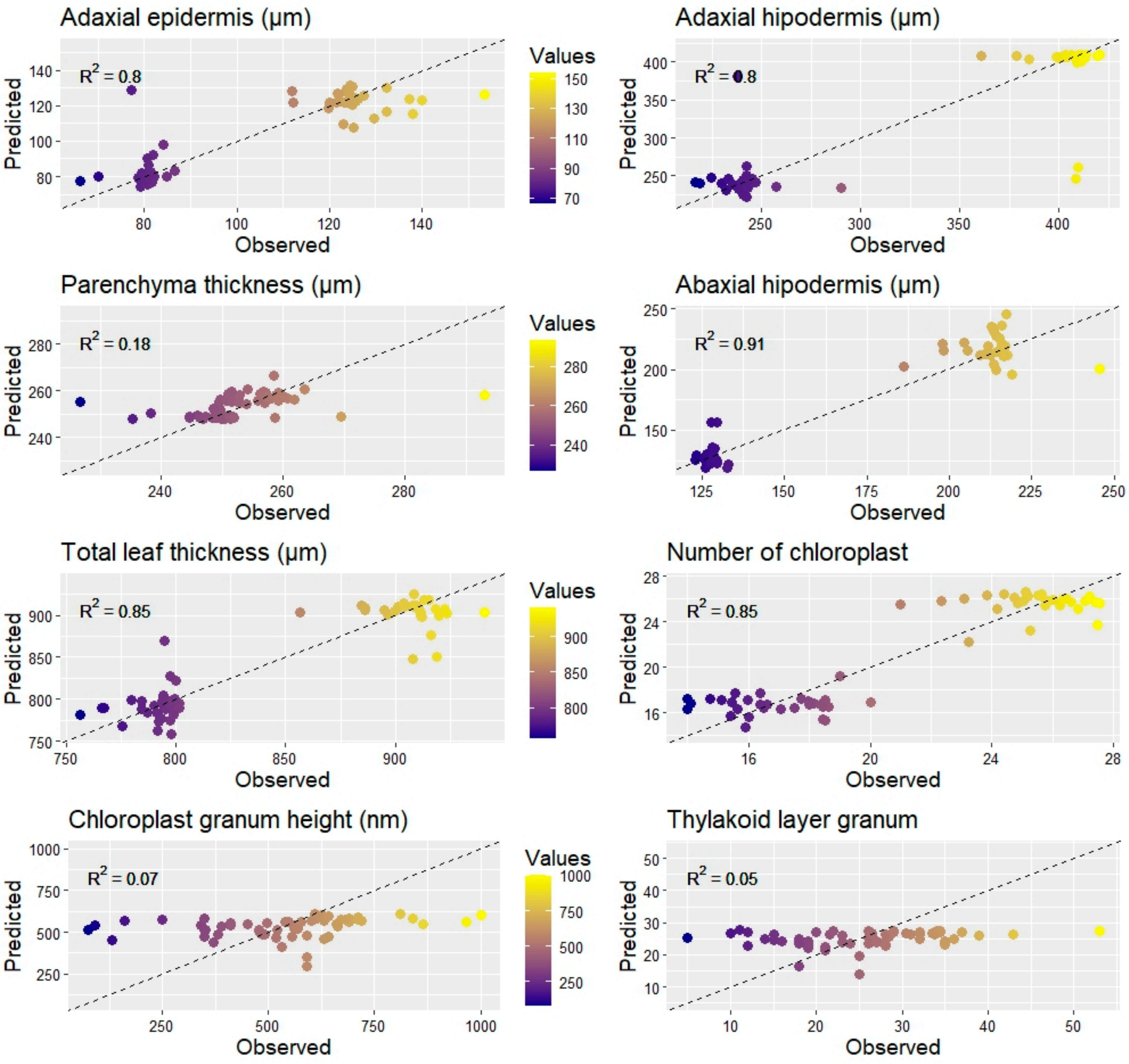

Figure S6. The observed vs. predicted data were estimated using partial least squares regression (PLSR) with hyperspectral reflectance data. (*n* = 50).

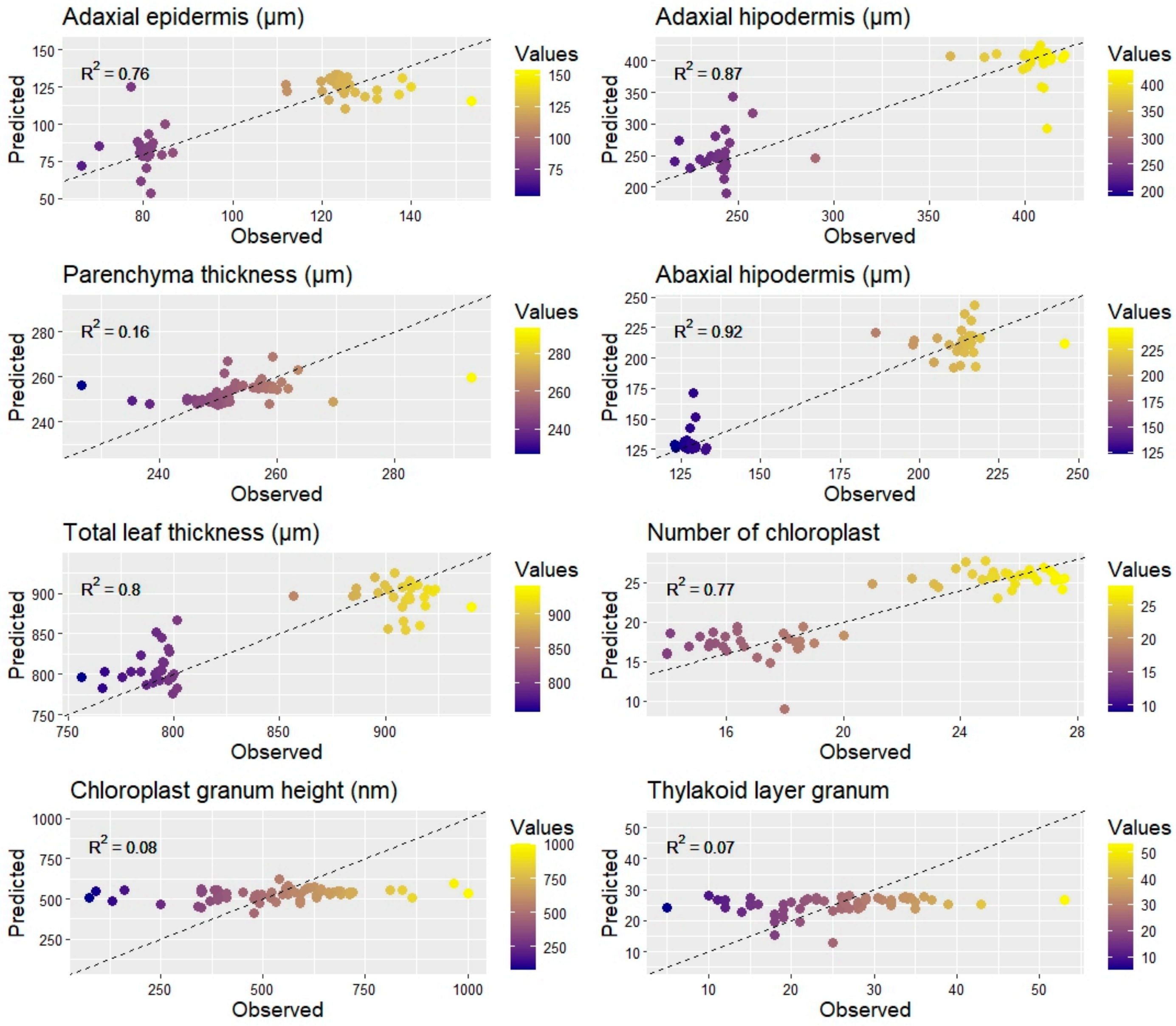

Figure S7. The measured data were compared to the data calculated via partial least squares regression (PLSR) with hyperspectral transmittance data. (n = 50).

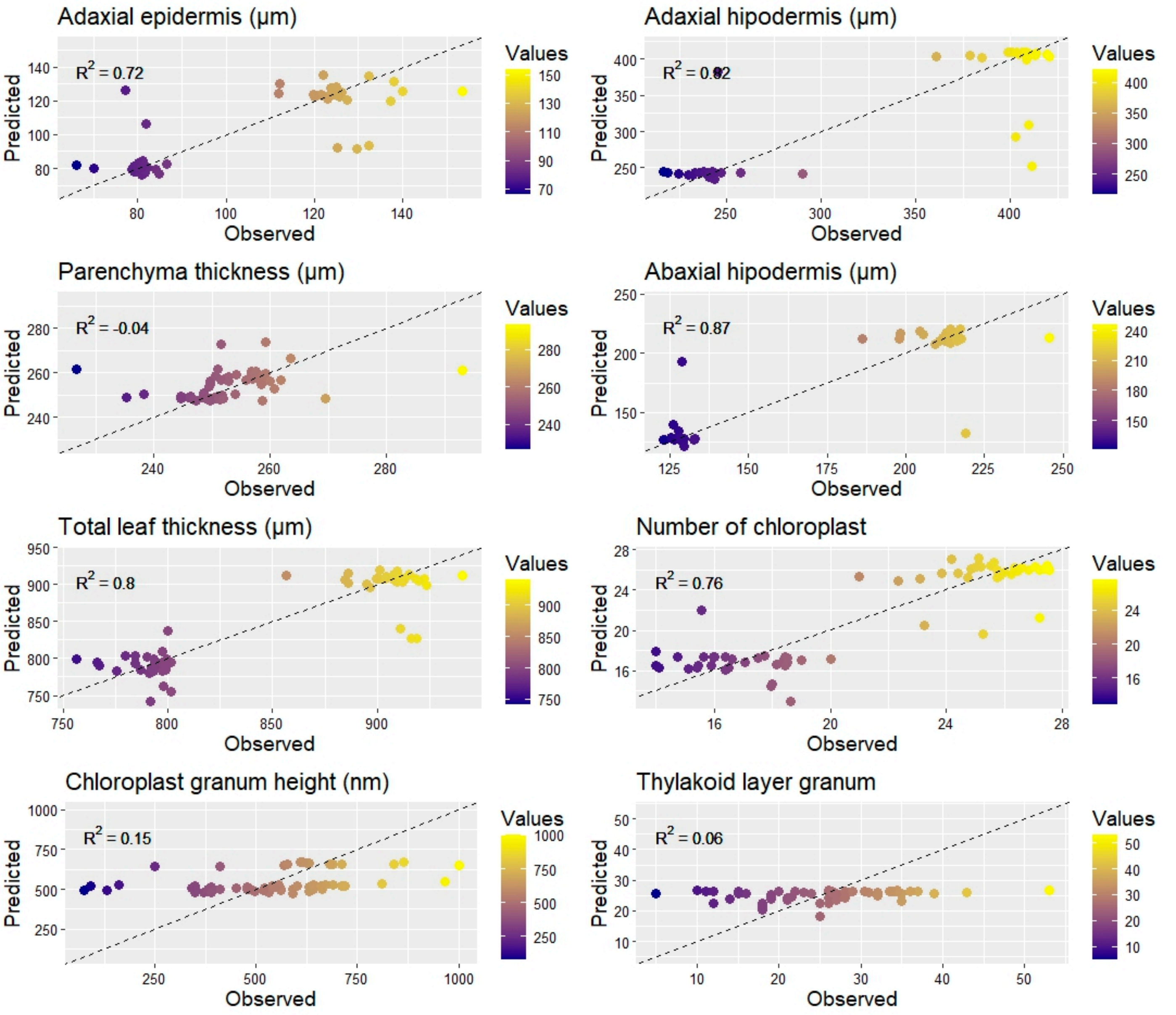

Figure S8. Observed vs. predicted data estimated by partial least squares regression (PLSR) of hyperspectral absorbance data. (n = 50).
